# Comparison of the Effects of Pilates and Yoga Exercise on the Dynamic Balancing Ability and Functional Movement of Fencers

**DOI:** 10.3390/life14050635

**Published:** 2024-05-16

**Authors:** So-Jung Lim, Hyun-Jin Kim, Yong-Soo Kim, Eunkuk Kim, Inyoung Hwang, Ju-Seop Kang

**Affiliations:** 1 Exercise Physiology Lab, Department of Physical Education, Graduate School, Korea University, Seoul 02841, Republic of Korea; koreasojung@korea.ac.kr; 2 Department of Pharmacology, College of Medicine, Hanyang University, Seoul 04763, Republic of Korea; hope0211@hanyang.ac.kr; 3 Department of Physical Education, Korea National Sport University, Seoul 05541, Republic of Korea; kys610@knsu.ac.kr; 4 SRC Rehabilitation Hospital, Gwangju 62421, Gyeonggi-do, Republic of Korea; 5 Department of Clinical Pharmacology and Therapeutics, Hanyang University Hospital, Seoul 04763, Republic of Korea; isoleucine@hanyang.ac.kr

**Keywords:** fencing, injury prevention, Pilates exercise, yoga exercise, LQ-YBT, FMS

## Abstract

This study was conducted to compare and analyze whether Pilates exercise and yoga exercise help improve the performance of female fencers and prevent injury, and the dynamic balance test (LQ-YBT) and functional movement screening (FMS) test score of the elite adult female fencers were compared and analyzed as evaluation indicators. Participants were randomly classified into Pilates (*n* = 10) and yoga groups (*n* = 10), members of which took part in 50 min of exercise (5 min of warm-up, 40 min of main exercise, and 5 min of cool-down) twice weekly for eight weeks. The results obtained from this study were analyzed via independent *t*-test and 2-way ANOVA. The results were as follows: LQ-YBT measures (reaching distance) increased significantly for both groups, as did FMS scores (deep squat, hurdle step, inline lunge, shoulder mobility, active straight-leg raise, trunk-stability push-up, and rotary stability). These results suggest that Pilates exercise and yoga exercise might be likely effective in improving the performance of adult female fencers and injury prevention by increasing their dynamic balance ability and functional movement.

## 1. Introduction

Fencing is a combat sport in which points are earned by attacking the opponent’s body [1]. It requires a high level of strategic thinking, agility, instantaneous reflexes, and the ability to make rapid changes in direction and braking movements. For this purpose, dynamic neuromuscular control ability is required. This is the ability to maintain stability while moving quickly, reacting to attacks, performing defensive maneuvers, and changing the direction of movement [2]. High-speed and instantaneous reflexive movements require fencers to use skeletal muscles explosively, which increases the risk of joint and muscle injuries [3]. Due to the need to perform repetitive unilateral movements, unbalanced movement patterns may occur, and weakness and injuries are common [4]. Fencers frequently suffer from ankle and knee injuries while making repetitive unilateral and excessive movements, and these injuries can lead to a serious decrease in performance [2]. Many fencers complain of anterior knee pain, and proper knee alignment and strong muscles help maintain knee stability and prevent injury [5]. A basic requirement when performing any of the technical movements of fencing, including those of attack and defense, is maintaining body balance. The ability to efficiently coordinate the body is closely related to athletic performance, and fencing requires accurate recognition of the position of the sword arm and the appropriate balance of hand and foot skills [5]. Imbalance in the body interferes with correct movements, deforms movement patterns through compensation, and induces deterioration of functional movement [6]. Eventually, this can lead to injury due to a decrease in proprioception [7].

Preventing injuries and improving performance among athletes, and fencers in particular, require more research. Injuries are critical risk factors for improving and maintaining athletic performance [8]. It is also more important to prevent injury than to rehabilitate after suffering an injury [9], and relevant research can ensure the health and safety of players and promote optimal performance. Research into the development of exercise programs to stabilize joints and prevent injuries caused by muscle imbalance is being conducted [2,10,11]. Effective injury-prevention exercise programs typically include a combination of strength [11] and neuromuscular training, proprioception, and balance exercises designed to improve athletic skills and overall performance [2,12]. Numerous studies have shown that strength training can reduce the risk of sports injuries [13,14] and that balance training is beneficial in reducing the rate of injuries [10]. In addition, a study on effective sports-injury-prevention exercises found that proprioceptive exercise was effective in reducing sports injuries [15]. Exercises such as balance and neuromuscular routines can reduce ankle injuries by 31% to 46% [10,12]. The same studies provide evidence of a need for muscle strength, balance, and proprioception in injury-prevention programs. Core strength is particularly important in sports because it provides “proximal stability for distal mobility” [16].

Pilates is based on six basic principles: breathing, control, accuracy, centralization, concentration, and flow [17]. Ballet and dance programs have long used Pilates to prevent injuries, and it has been employed to rehabilitate injured athletes [17,18,19,20] in various sports, such as golf and archery [21,22]. It reportedly improves movement control, balance, stability, and flexibility and strengthens core muscles [17,21]. Numerous studies have shown that muscle imbalance and decreased trunk stability are closely related to injuries [23,24]. Exercises to improve trunk stability by strengthening core muscles are necessary. However, strengthening exercises in local areas alone can lead to physical imbalance and decreased performance [25]. Small muscles in the body can be strengthened through contraction of the transversus abdominis, pelvic floor, and internal and external oblique muscles and through relaxation of the diaphragm [21,22,26]. Unilateral athletes need training routines, such as Pilates [26], that connect movements of the upper and lower extremities based on trunk stability [27].

Yoga, another type of exercise, improves muscle tension, helps improve flexibility by stretching atrophied muscles, and serves as a stabilization exercise that strengthens trunk muscles [28,29,30]. Breathing and posture training reportedly promotes mental stability, alleviates imbalances between the body and mind, improves muscle strength and flexibility, and protects the body from stress-related diseases [31]. Multiple studies have shown that yoga can improve mobility and trunk stability and demonstrated the importance of the effective design and management of injury-prevention training [32,33]. Such studies are important reference materials for research on injury prevention and performance improvement in fencers.

We planned to compare and determine whether there were any changes in dynamic balance ability and functional movement before and after engaging in Pilates and yoga exercises designed for fencers, and whether there was a difference in functional movement and dynamic balance ability between Pilates and yoga exercisers.

This study was conducted to compare and analyze whether Pilates exercise and yoga exercise help improve the performance of female fencers and prevent injury, and the dynamic balance test (LQ-YBT) and functional movement screen (FMS) score of the elite adult female fencers were compared and analyzed as evaluation indicators.

## 2. Materials and Methods

### 2.1. Participants

This study was conducted on adult female fencers (with more than 5 years of experience) aged 18–22 years old in Seoul, South Korea, who were registered as athletes with the Korean Fencing Federation and who had not had any surgery within the past 6 months, regardless of physical differences (Table 1). The purpose, methods, and procedures of this study were explained in detail to participants. Those who had undergone surgery within the previous 6 months were excluded. An explanation of the physical changes, muscle pains, and safety accidents that might occur during the experiment was offered to all participants, who provided written informed consent, and the study was approved by the Research Ethics Committee of Korea National Sport University (Protocol No. 20200612-068). The characteristics of the study subjects are shown in Table 1.

### 2.2. Research Design (Program and Process)

We hypothesized that fencers in the two groups (Pilates and yoga) would experience several changes after an 8-week exercise program. First, there would be changes in the left-and right-foot reach distances of the lower-extremity dynamic balance as measured by the lower-quarter Y-balance test (LQ-YBT) and the functional movement screening (FMS^TM^) test, which consists of stability scores for overhead deep squat (ODS), hurdle step (HS), inline lunge (IL), shoulder mobility (SM), active straight-leg raise (ASLR), trunk-stability push up (TSP), and trunk rotation (TR) exercises. Second, we expected that the effect of Pilates would be greater than that of yoga. The purpose of this study is to provide basic data to prevent injuries and improve performance in fencers by comparing and analyzing the effects of Pilates or yoga on dynamic balance ability and functional movement.

**Figure 1:**
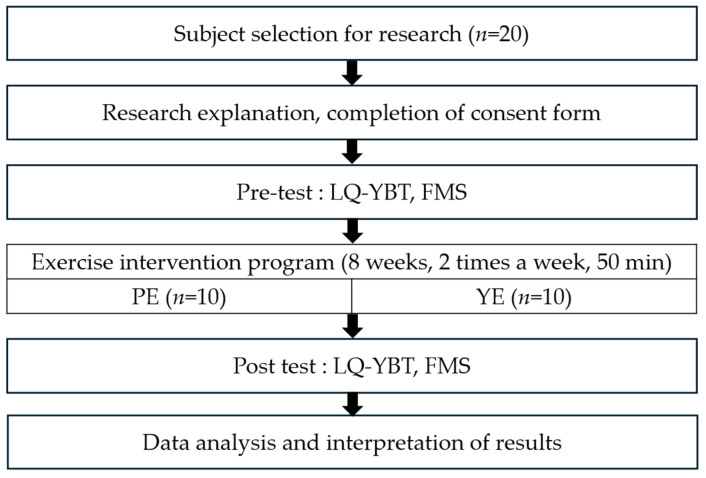
Flow diagram of study processes: lower-quarter Y-balance test and functional movement screen.

For the 20 female fencers who participated in the study, we measured anterior (AT), posteromedial (PM), and posterolateral (PL) values of the LQ-YBT to evaluate dynamic balance ability, and then measured ODS, HS, IL, SM, ASLR, TSP, and RS values of the FMS to evaluate functional movement. After participating in the existing training of their fencing club, the participants were randomly divided into assigned groups and performed the exercise program presented in Figure 1 and Table 2.

#### 2.2.1. Pilates Exercise Program

The Pilates exercise program was composed of basic and intermediate movements based on a literature review and expert opinion [34,35]. It consisted of 5 min of warm-up, 40 min of main exercise, and 5 min of cool-down exercise to strengthen coordination and trunk stability. It was performed twice a week for 8 weeks. The instructor held a Pilates professional instructor certification and had more than 10 years of teaching experience. Using Borg’s subjective scale [36], the exercise intensity was gradually increased by dividing it into 1 to 4 weeks and 5 to 8 weeks (Table 2).

#### 2.2.2. Yoga Exercise Program

The yoga exercise program was composed of basic and intermediate movements based on a literature review and expert opinions to improve the flow of consciousness and to develop and stabilize whole-body balance and flexibility through breathing and meditation [30,37]. The yoga exercise program included 5 min of warm-up, 40 min of main exercise, and 5 min of cool-down, followed by 5 min of meditation. The instructor held a professional yoga instructor certification and had more than 10 years of teaching experience. The intensity of exercise was gradually increased by dividing it into 1 to 4 weeks and 5 to 8 weeks using Borg’s subjective scale [36] (Table 2).

### 2.3. Measurements

#### 2.3.1. Y Balance Test (Lower-Quarter Y-Balance Test)

**Figure 2:**
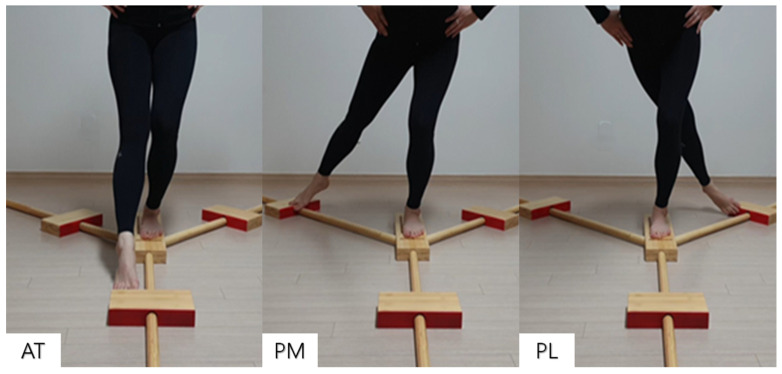
Lower-quarter-Y balance test: AT, anterior; PM, posteromedial; PL, posterolateral.

The Y-Balance Test Kit (FMS Inc., Chatham, VA 24531, USA) was used to measure the dynamic balance of the lower extremities (Figure 2). To measure lower-limb length, participants were asked to lie supine on a bed, and the distance from the anterior superior iliac spine to the medial malleolus of the ankle was measured using a tape measure. All participants watched a video that provided an explanation and demonstration of the LQ-YBT measurement method and a researcher’s demonstration. After familiarization by practicing twice each before the measurement, the main test was performed. The participants then took off their shoes, stood with the tip of one foot aligned with the footrest of the equipment, and assumed a posture with the pelvis resting on both hands. While keeping the entire sole of the supported foot off the floor, participants were advised to be careful not to bend the upper body excessively forward and to fully extend the feet in anterior, posteromedial, and posterolateral directions. Failure to return to the starting position, loss of balance, kicking the footrest, or touching the ground was considered a foul [38]. This was performed on both the left and right sides, and after practicing twice each, the longest stretching distance was measured 3 times in each direction. The reaching distance in a total of 6 directions, consisting of the anterior, posteromedial, and posterolateral directions on the right and left, was measured, and the score for each direction was calculated by multiplying the longest reaching length. After dividing by 3 times the length of the lower extremity (the length from the anterior superior iliac spine to the medial malleolus), the result was multiplied by 100.

#### 2.3.2. Functional Movement Screen

FMS also provided explanations and demonstrations of the measurement method (researcher’s demonstration), and because reliability could be overestimated due to memory effects, the highest score was adopted after measuring twice. As a functional movement evaluation tool to evaluate flexibility, mobility, and stability [39], the FMS test presents standards for evaluating 7 basic movement patterns (Table 3) and gauges the movements of each stage to improve the body’s performance. It was designed to effectively identify functional limitations and imbalances. Pain is scored as 0, and each movement is given 1, 2, or 3 points. Coordination was judged as the sum of 7 items, with the lowest score selected for both measurements [7,40].

### 2.4. Statistical Analysis

For analysis of our data, descriptive statistics were calculated in SPSS version 25.0, and as both the group FMS and LQ-YBT scores a satisfied normal distribution, an independent *t*-test was conducted. A paired *t*-test was then conducted to confirm changes in the FMS and LQ-YBT scores before and after exercise within the group. In addition, the variables of each group satisfied the homogeneity test, and a two-way repeated-measures analysis of variance was conducted to confirm the interaction effect between the groups and measurement times. All statistical significance levels were set at alpha = 0.05.

## 3. Results

**Figure 3:**
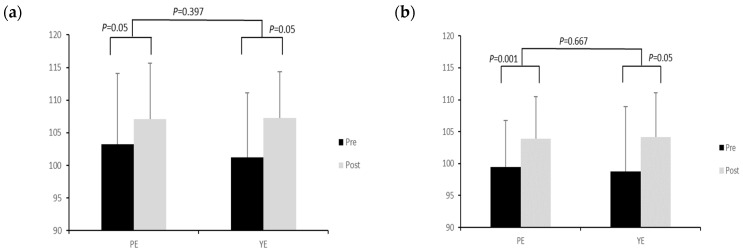
Changes in lower-limb LQ-YBT composite scores according to exercise between groups. (**a**) Left-foot variation in LQ-YBT; (**b**) Right-foot variation in LQ-YBT.

In the group-specific exercise-based LQ-YBT, the left-foot reaching distance of the LQ-YBT in the Pilates exercise group increased significantly after the pre-test, and the yoga exercise group also significantly increased the left-foot reaching distance of the LQ-YBT after the pre-test (Figure 3). No statistically significant difference was seen in the timing and interaction effects between the groups.

Based on the FMS scores, functional movement increased significantly after both Pilates and yoga exercises, but no significantly different pre–post effect was detected between the groups. Looking at the changes in each item, such as the ODS, HS, IL, SM, ASLR, TSP, and TR, the stability scores increased significantly, showing that Pilates and yoga helped improve dynamic balance and functional movement (Table 4).

## 4. Discussion

The results showed that Pilates and yoga significantly improved the dynamic balance ability as measured by LQ-YBT and FMS scores compared with before exercise.

As previously reported, similar improvements were seen in the balance ability of archers after 12 weeks of Pilates exercise [21], and balance and flexibility ability were significantly improved in college students after 10 weeks of yoga [37]. In these reports, both exercises were conducted with a focus on core stabilization, balance ability, and coordination. According to the current literature, muscle imbalance and decreased core stability and balance due to unilateral exercise can increase the injury rate in athletes [12,15,16,41]. Injuries can seriously affect an athlete’s performance and career. However, athletic training often focuses only on improving physical strength and does not sufficiently consider injury prevention. It is therefore necessary to incorporate elements of injury prevention into training programs. The LQ-YBT is a lower-extremity dynamic-balance-ability and neuromuscular-control evaluation tool designed to help demonstrate an individual’s ability to balance the body at the limit of stability [38,42]. It is used to measure athletes’ dynamic balance ability and left–right balance and to predict injuries in athletes [38]. The LQ-YBT is based on a subset of reaching directions (AT, PL, and PM) that are part of the Star Excursion Balance Test (SEBT) and has a favorable inter-rater reliability (0.99–1.00) and an excellent intra-rater reliability (intraclass correlation = 0.85–0.91) [43]. The LQ-YBT has a shorter and simpler measurement time than the SEBT, and it is widely used as a measurement method in the field. The LQ-YBT has repeatedly demonstrated good inter-rater and intrarater reliability [44].

Approximately half of all injuries among fencers occur in the ankles and knees due to repetitive unilateral movements and excessive panting movements, with the most frequent injury being ankle sprain [2]. Sports-related injuries are one of the most important risk factors affecting athletic performance [8]. These injuries can lead to changes in neuromuscular and proprioceptive function and posture, as well as a decrease in dynamic neuromuscular control, which can, in turn, lead to diminished athletic performance and various injuries [45].

Pilates is a mind–body exercise that focuses on strength and muscle control, core stability, postural control, flexibility, and breathing. Pilates and yoga are thought to have a positive effect on dynamic balance ability, but the ability to determine dynamic balance ability using only the LQ-YBT score is limited. The injury-prediction rate can be increased by performing an FMS test [16,46]. Additional research is needed on the impact of improving dynamic balance ability on injury prevention. No statistically significant difference in the timing and group-interaction effects between the Pilates and yoga exercise groups was seen in our study.

In both the Pilates and yoga groups, the post-FMS score increased statistically significantly after exercise compared with before exercise, with a significant improvement in functional movement evident. This confirms previous research showing that Pilates and yoga had a positive effect on the functional movement and personal health of exercise participants [47], and a study by Jung et al. that also targeted active females. The effectiveness of the exercise was verified by dividing the subjects into Pilates exercise and trunk-stability groups, for which the FMS score and LQ-YBT scores improved [48]. In addition, the effect of yoga on the functional movement, dynamic balance ability, and trunk stability of 43 adults showed that yoga, Pilates, and core exercise were effective in treating adults with chronic lower-back pain [48]. They have also been shown to be helpful in improving functional movement, dynamic balance ability, and trunk stability.

The FMS has high reliability, and low FMS performance has been confirmed to be closely related to injury risk in professional football players, military personnel, female college athletes, and fire fighters [46,49], while an increase in FMS score reduced injuries in ballet dancers [23]. A positive effect on injury prevention has also been reported. Based on these results, Pilates and yoga are believed to be helpful in improving the dynamic balance ability and functional movement of fencers. An injury-prevention program including Pilates and yoga exercises for 8 weeks combined with physical and technical training should therefore have a positive effect in preventing injuries and improving performance [50] in fencers. Future studies will be needed to identify any correlation between improvements in dynamic balance ability on the one hand and functional movement and injury prevention and exercise performance on the other [50].

Because this paper is limited to adult female athletes aged 18 to 24 years, multifaceted research on various population groups (e.g., children, adolescents, and adult males) is needed. The FMS test is a useful tool for evaluating athletic performance and functional impairments, such as joint mobility, coordination, and proprioception related to injuries, but it does not take into account the characteristics of the sport and age, and only evaluates athletic performance with the FMS score. There may be limitations in predicting injury rates [51,52]. Research needs to be conducted on how the scores obtained through FMS evaluations contribute to the injury rate and performance of players. Studies are needed that examine options for preventing injuries and improving skills [50] and for developing injury-prevention measurement methods that take into account the unique characteristics of fencing and that monitor physical functions related to major injuries. Research should also be conducted to verify the effectiveness of injury-prevention exercise programs using large samples of various age groups and genders.

In this study, after performing Pilates and yoga exercises, dynamic balance ability and functional movement were significantly improved, suggesting that Pilates and yoga may help prevent injuries and improve performance in fencers. However, because there was no statistically significant difference between the two exercise groups, both Pilates and yoga appear to be useful, and the choice of exercise regime should be based on the athlete’s circumstances and preferences. In future research, we propose to attempt to predict major injury areas in fencers and to determine the effectiveness of sport-specific injury prevention and skill-improvement programs for subjects of various ages and genders.

Limitations of this study included that it only targeted 20 female athletes aged 18 to 22 years; did not control for lifestyles, psychological factors, or physical activity other than the subjects’ exercise programs; and did not distinguish between playing seasons and off-seasons. Also, because the LQ-YBT and FMS are complex and influenced by many factors, it is not easy to draw a conclusion based on only one study result, and more extensive research and analysis is needed.

## 5. Conclusions

Pre-training with Pilates and yoga exercises may similarly contribute to improvements in the dynamic balance ability and functional movement of fencers, which would help to prevent injuries and improve athletic performance. Therefore, both Pilates and yoga appear to be useful, and the choice of exercise regime should be based on the athlete’s personal circumstances and preferences.

## Figures and Tables

**Table 1:** Baseline participant and demographic characteristics (*n* = 20).

Variable *	Pilates Exercise Group	Yoga Exercise Group	*p*-Value
Age (years)	21.00 ± 0.82	19.50 ± 1.27	0.008
Height (cm)	163.00 ± 5.57	169.27 ± 7.14	0.042
Body weight (kg)	59.71 ± 6.29	63.64 ± 9.03	0.274
BMI (kg/m^2)^	22.49 ± 2.12	22.18 ± 1.79	0.728
TBF (%)	25.77 ± 5.62	23.95 ± 3.48	0.395

* Values are expressed as the mean ± standard deviations. BMI = body mass index; TBF = total body fat. Significant differences between groups are indicated by *p* < 0.05.

**Table 2:** Exercise intervention program.

Group	Pilates Exercise	Yoga Exercise	Intensity (RPE *, Unit)		Time (min)
			1–4 Weeks	5–8 Weeks	
Warm- up	Breathing, stretch, pelvic tilt	Breathing, stretch, Suryanamaskar	8–10	8–10	5
Main exercise	Basic: The hundred, roll up and down, single leg circle, criss-cross, single/double leg stretch	Basic: Sitting pose, child pose, camel pose, down-dog pose, tree pose, cobra pose	11–13	13–15	40
	Intermediate: Side-kick series, single/double straight leg, teaser	Intermediate: Warrior pose			
	swimming, seal, shoulder bridge, plank, side plank, open-leg rocker	Extended- triangle pose, fish pose, eagle pose, boat pose, plank pose, low-plank pose			
Cool- down	Full-body stretch, rest position	Full-body stretch, meditation	8–10	8–0	5

* RPE (rating of perceived exertion): The Borg rating of the perceived exertion scaling system (level of exertion 1–20).

**Table 3:** Photographs and descriptions of the participants in the functional movement screen scoring system [7,40].

Tests	Score	Scoring Criteria
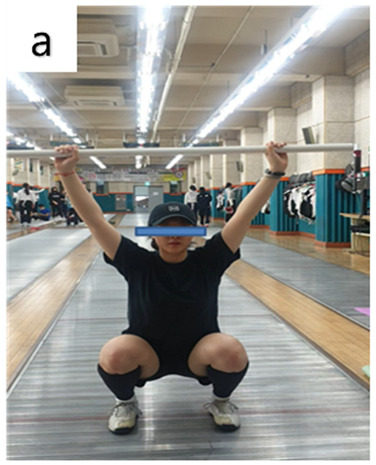	3	The upper torso is parallel with the tibia or toward the vertical, the femur is below the horizontal, the knees are aligned over the feet, and the dowel is also aligned over the feet
	2	The upper torso is parallel with the tibia or toward the vertical, the femur is below the horizontal, the knees are over the feet, and tthe dowel is also aligned with the feet; however, the heels are elevated on a 2-inch board.
	1	The tibia and the upper torso are not parallel, the femur is not below he horizontal, the knees are not aligned over the feet, or lumbar flexion is noted. Heels are elevated on a 2-inch board.
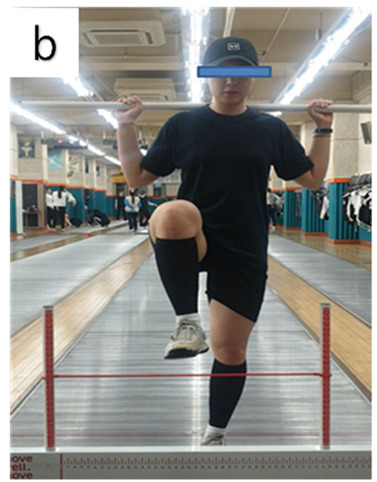	3	Hips, knees, and ankles remain aligned in the sagittal plane. Minimal to no movement is noted in the lumbar spine, and the dowel and hurdle remain parallel.
	2	Alignment is lost between the hips, knees, and ankles. Movement is noted in the lumbar spine, or the dowel and hurdle do not remain parallel.
	1	An athlete must be scored as a “1” if contact with the hurdle occurs during the test, or if a loss of balance is noted.
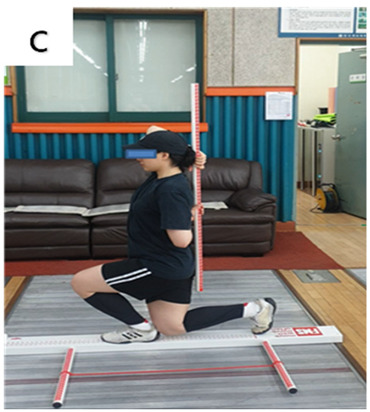	3	The dowel remains vertical and in contact with the spine, there is no torso movement noted, the dowel and feet remain in the sagittal plane, and the knee touches the board behind the heel of the front foot.
	2	Dowel contact is not maintained, the dowel does not remain vertical, movement is noted in the torso, the dowel and feet do not remain in the sagittal plane, or the knee does not touch behind the heel of the front foot.
	1	A score of “1” is awarded if the athlete loses balance.
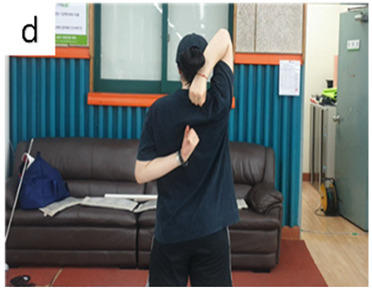	3	Fists are within one hand-length.
	2	Fists are within one and one-half hand-lengths.
	1	Fists are not within one and one-half hand-lengths.
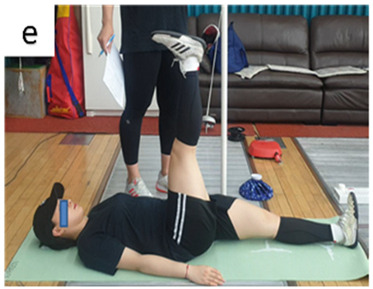	3	The vertical line of the malleolus of the tested leg resides between the mid-thigh and the anterior superior iliac spine. The non-moving limb must remain in a neutral position.
	2	The vertical line of the malleolus of the tested leg resides between the mid-thigh and the knee joint line. The non-moving limb must remain in the neutral position.
	1	The vertical line of the malleolus of the tested leg resides below the knee joint line. The non-moving leg must remain in the neutral position.
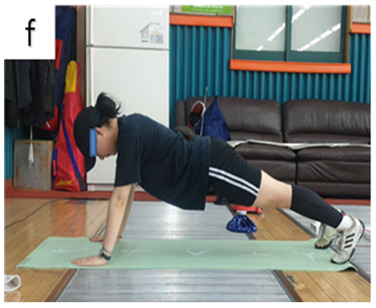	3	The body lifts as a unit with no lag in the spine. Men perform a repetition with thumbs aligned with the top of the head; women perform a repetition with thumbs aligned with the chin.
	2	The body lifts as a unit with no lag in the spine. Men perform a repetition with thumbs aligned with the chin; women perform a repetition with thumbs aligned with the clavicle.
	1	A score of “1” is given if the subject is unable to perform a repetition (with the body lifting as a unit) in the hand positions (men’s thumbs aligned with the chin; women’s with the clavicle).
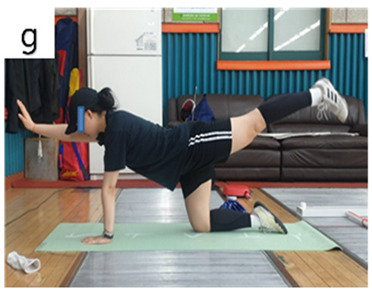	3	The subject performs a correct unilateral repetition. A. Extended position (does not have to be >6–8 inches off the ground). B. Flexed position; elbow and knee must meet. Note: Must maintain narrow upper- and lower-extremity weight bearing over the 2 × 6 inch board without a major weight shifting away from the board.
	2	The subject performs a correct diagonal repetition. A. Extended position (does not have to be >6–8 inches off the ground). B. Flexed position; elbow and knee must meet. Note: Must maintain narrow upper- and lower-extremity weight bearing over the 2 × 6 inch board without a major weight shift away from the board.
	1	The subject is unable to perform a diagonal repetition. A. Extended position. B. Flexed position.

a, deep squat; b, hurdle step; c, inline lunge; d, shoulder mobility; e, active straight-leg raise; f, trunk-stability push-up; g, rotary stability test.

**Table 4:** Statistical analysis of the change in the FMS score before and after exercise between and within groups.

Test/Group/Time		Pre	Post	WG	ANOVA
a	PE	1.30 ± 0.48	2.30 ± 0.67	*t =* −4.743; *p* < 0.01 **	*F* = 0.474 *p* = 0.500
	YE	1.20 ± 0.42	2.00 ± 0.82	*t =* −4.000; *p* < 0.01 **	
	BG	*t* = 0.493; *p =* 0.628	*t* = 0.896; *p* = 0.382		
b	PE	1.50 ± 0.53	2.60 ± 0.52	*t =* −11.000; *p <* 0.001 ***	*F =* 0.360 *p =* 0.556
	YE	1.20 ± 0.42	2.40 ± 0.52	*t = −*9.000; *p <* 0.001 ***	
	BG	*t =* 1.222; *p =* 0.237	*t* = 0.866; *p =* 0.398		
c	PE	1.50 ± 0.53	2.80 ± 0.42	*t = −*6.091; *p <* 0.001 ***	*F* = 2.057 *p =* 0.169
	YE	1.50 ± 0.53	2.40 ± 0.52	*t = −*5.014; *p <* 0.01 **	
	BG	*t =* 0.000; *p* = 1.000	*t =* 1.897; *p =* 0.074		
d	PE	1.20 ± 0.42	1.70 ± 0.67	*t = −*3.000; *p <* 0.05 *	*F =* 0.545 *p =* 0.470
	YE	1.20 ± 0.42	1.90 ± 0.57	*t = −*3.280; *p <* 0.05 *	
	BG	*t* = 0.000; *p* = 1.000	*t =* −0.717; *p =* 0.482		
e	PE	1.60 ± 0.70	2.50 ± 0.71	*t = −*3.857; *p <* 0.01 **	*F =* 0.101 *p =* 0.754
	YE	1.50 ± 0.71	2.50 ± 0.53	*t = −*4.743; *p <* 0.01 **	
	BG	*t* = 0.318; p = 0.754	*t* = 0.000; *p* = 1.000		
f	PE	1.20 ± 0.42	1.90 ± 0.74	*t = −*3.280; *p <* 0.05 *	*F =* 0.545 *p =* 0.470
	YE	1.10 ± 0.32	1.60 ± 0.70	*t = −*3.000; *p <* 0.05 *	
	BG	*t =* 0.600; *p =* 0.556	*t =* 0.933; *p =* 0.363		
g	PE	1.30 ± 0.48	2.70 ± 0.67	*t = −*6.332; *p <* 0.001 ***	*F =* 0.106 *p =* 0.749
	YE	1.30 ± 0.48	2.60 ± 0.52	*t = −*6.091; *p <* 0.001 ***	
	BG	*t* = 0.000; *p* = 1.000	*t =* 0.372; *p =* 0.714		
h	PE	9.60 ± 1.58	16.50 ± 2.17	*t = −*14.318; *p <* 0.001 ***	*F =* 0.106 *p =* 0.749
	YE	9.00 ± 1.41	15.40 ± 1.84	*t = −*9.798; *p <* 0.001 ***	
	BG	*t* = 0.896; *p =* 0.382	*t =* 1.222; *p =* 0.237		

Abbreviations: Pilates exercise: PE; yoga exercise: YE; pre-test: pre; post-test: post; between group: BG; WG: within group; (a) overhead deep squat: ODS; (b) hurdle step: HS; (c) inline lunge: IL; (d) shoulder mobility: SM; (e) active straight-leg raise: ASLR; (f) trunk-stability push-up: TSP; (g) rotary stability: RS; (h) FMS total score: FTS. * Values are expressed as the mean ± standard deviation *(n* = 20), * *p* < 0.05, ** *p* < 0.01, *** *p* < 0.001.

## Data Availability

Data are contained within the article.
